# Association of proinflammatory genes expression with serum interleukin 1β and free fatty acids in metabolically healthy and unhealthy abdominally obese individuals: a case-control study

**DOI:** 10.1186/s12865-019-0303-2

**Published:** 2019-07-04

**Authors:** Parichehr Amiri, Behzad Baradaran, Maryam Saghafi-Asl, Mahsa Naghizadeh, Dariush Shanehbandi, Nahid Karamzad, Sepideh Zununi Vahed

**Affiliations:** 1 Student Research Committee, Tabriz University of Medical Sciences, Tabriz, Iran; 2 Immunology Research Center, Tabriz University of Medical Sciences, Tabriz, Iran; 3 Department of Clinical Nutrition, School of Nutrition and Food Sciences, Tabriz University of Medical Sciences, Tabriz, Iran; 4 Department of Public Health, School of Nursing and Midwifery, Maragheh University of Medical Sciences, Maragheh, Iran; 5 Kidney Research Center, Tabriz University of Medical Sciences, Tabriz, Iran

**Keywords:** Toll like receptor2, Myeloid differentiation Factor88, Nuclear factor kappa B, interleukin-1beta, Abdominal obesity

## Abstract

**Background:**

Proinflammatory genes are highly expressed in several metabolic disorders associated with obesity. But it is not clarified whether gene expression levels and downstream inflammatory markers are related to the metabolic state or the presence of obesity. Hence, the present study aimed to compare Toll-Like Receptor 2 (TLR2), Myeloid Differentiation Factor 88 (MyD88), and NFĸB mRNA expression levels between metabolically healthy abdominally obese (MHAO) and metabolically unhealthy abdominally obese (MUAO) individuals.

**Results:**

We compared mRNA expression levels of the genes as well as serum FFAs and IL-1β in MUAO (*n* = 36) and MHAO (*n* = 34) groups. Serum FBS, TG, and HDL-C in addition to systolic and diastolic blood pressure were significantly higher in MUAO than MHAO groups (*p* < 0.05). The odds of MUAO was significantly decreased with high HDL-C (OR = 0.22, 95%CI: 0.08–0.63) and increased with high FBS (OR = 7.04, 95%CI: 1.42–34.69) and TG (OR = 30.55, 95%CI: 7.48–60.67). There were no significant differences in proinflammatory genes as well as serum FFAs and IL-1β between the two groups. No associations were found between the genes expression and serum markers. However, NFĸB expression was significantly correlated with TLR2 and MyD88 (r = 0.747; *p* < 0.001). Significant correlations were also noticed between TLR2 and MyD88 expression as well as between serum FFAs and IL-1β in each group (*p* < 0.001).

**Conclusion:**

Serum concentration of IL-1β, FFAs, and mRNA expression levels of TLR2, MyD88, and NFĸB may be resulted from abdominal obesity and not be related to the presence or absence of metabolic health.

## Background

Obesity is a major universal health threat. Worldwide obesity has more than doubled since 1980 and evidence suggest that 51% of the population will be obese by 2030 [1]. Metabolic disorders are one of the main deleterious health risks of obesity [2]. However, several studies show that there are individual differences in metabolic reactions of obesity; therefore, there are different subtypes of obesity [3, 4].

A subgroup of obese individuals who do not have metabolic complications of obesity, are called metabolically healthy obesity (MHO) [5–9]. It is estimated that 10–25% of the obese individuals are MHO [10]; however, there is no definite criteria to describe it [4]. This phenotype is mainly characterized by normal metabolic parameters such as serum lipid and glucose levels and blood pressure, despite elevated adiposity and body mass index (BMI) [9, 11]. Accumulating data indicate that inflammation is the linking point of adiposity and metabolic disorders, but there is no convincing explanation for differences found in metabolically healthy and unhealthy obese individuals [12]. While some studies claim that MHO subjects have lower inflammation levels which contribute to their favorable metabolic profile, other reports do not show normal inflammatory profile for MHO individuals [13].

It is unclear whether inflammation starts in obesity state; however, the activity of some pathways initiating with Toll-Like Receptors (TLRs) is established. Toll-like receptor2 (TLR2), as one of the members of these metabolic sensors, has received more attention [14]. It is a pattern recognition immune receptor which recruits numerous adaptor proteins of the myeloid differentiation factor 88 (MyD88)-dependent pathway, causing subsequent inflammatory responses via activation of NFĸB [15, 16]. TLRs mediate NFĸB activation, as a principal transcriptional factor, which initiate inflammatory cascade and controls the expression of various inflammatory cytokines [16]. Animal studies have revealed that deficiency of MyD88 can reduce expression of proinflammatory genes and cytokines such as IL-1β and tumor necrosis factor-α (TNF-α) [17, 18]. IL-1β, as a pro-inflammatory cytokine, has been shown to have important effects on fat mass, fat metabolism, and body mass and induce insulin resistance [19].

It is elucidated that TLR2 dysregulation during obesity translates a metabolic challenge into an inflammatory response and contributes to obesity-associated metabolic diseases [14]. However, little is known about its activity in different obesity subtypes; moreover, their relationship with metabolic abnormalities is yet to be clarified. Abdominal obesity is more harmful than any other type of obesity with higher total body fat [20]. Researchers have revealed that abdominal (visceral) fat excess is highly concerned with metabolic diseases [21, 22]. Also, increased adipose tissue mass because of free fatty acids (FFAs) released from enlarged and hypertrophied adipocytes is a crucial in the progression of inflammation. Hence, investigation of expression levels of TLR2 with its main adaptor protein and their association with inflammatory factors seems to be demanded.

Studies on persons with diabetes and metabolic syndrome (MetS) have reported upregulated levels of TLR2 and MyD88 genes [23–25]. In contrast, a study revealed no significant differences in gene expression levels of TLR2 in peripheral blood mononuclear cells (PBMCs) of MHO and metabolically unhealthy obesity (MUO) individuals [26]. In addition, another research indicated that expression of genes involved in inflammation had a similar alteration pattern in MHO and MUO persons [27]. There is a paucity of data about TLRs activity in MHO and MUO persons and published results are conflicting [26–28]. It is not verified that observed expression patterns are result from adiposity or related to individuals metabolic status. For this reason, our hypothesis was that changes in serum inflammatory factors and expression levels of the related genes are independent of metabolic abnormities and are only related to the adiposity. Therefore, we considered high waist circumference (WC) as a marker of adiposity and investigated TLR2, MyD88, and NFĸB gene expression levels and serum inflammatory factors in metabolically healthy abdominally obese (MHAO) and metabolically unhealthy abdominally obese (MUAO) individuals.

## Results

**Fig. 1:**
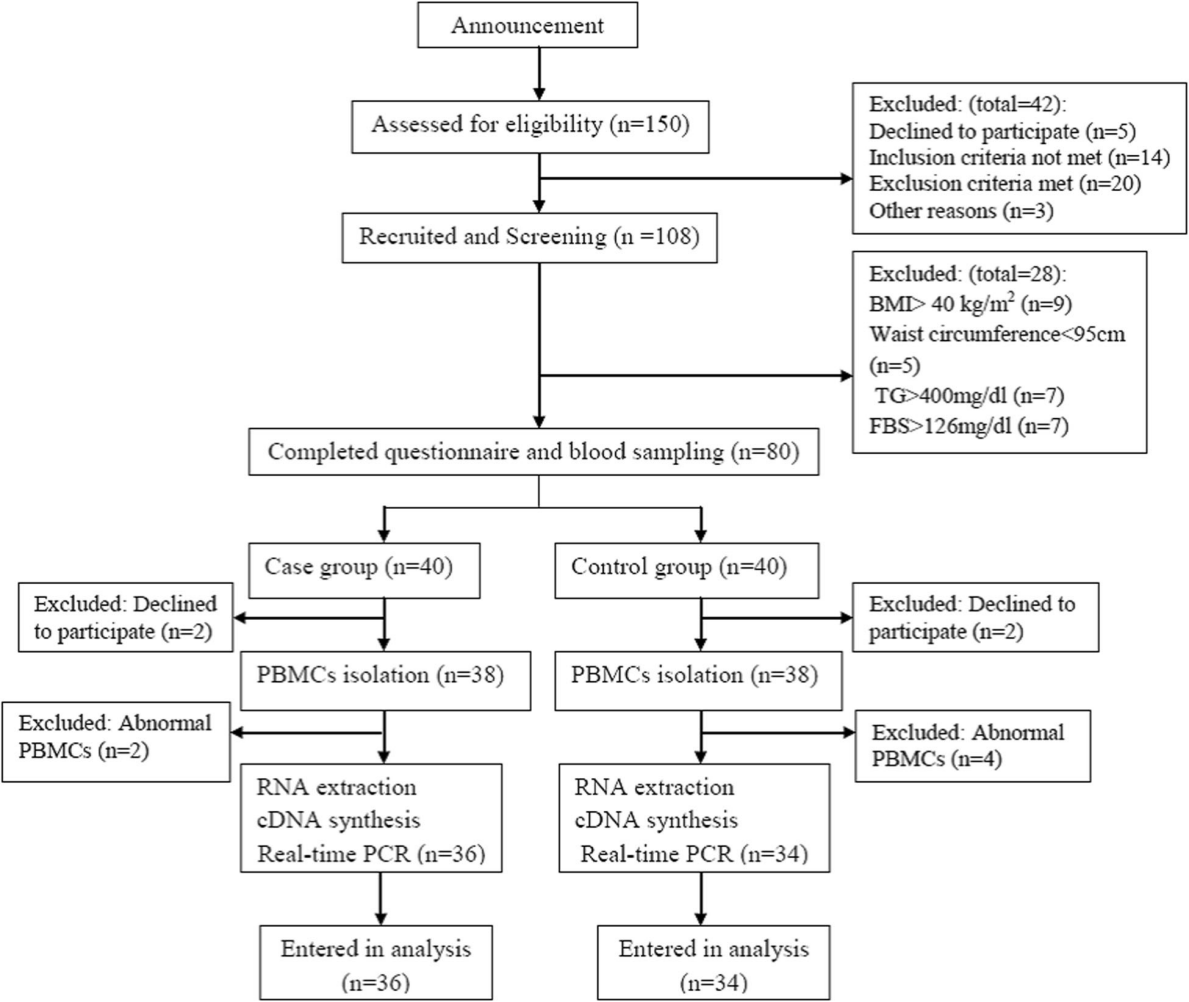
Flowchart of the study

**Fig. 2:**
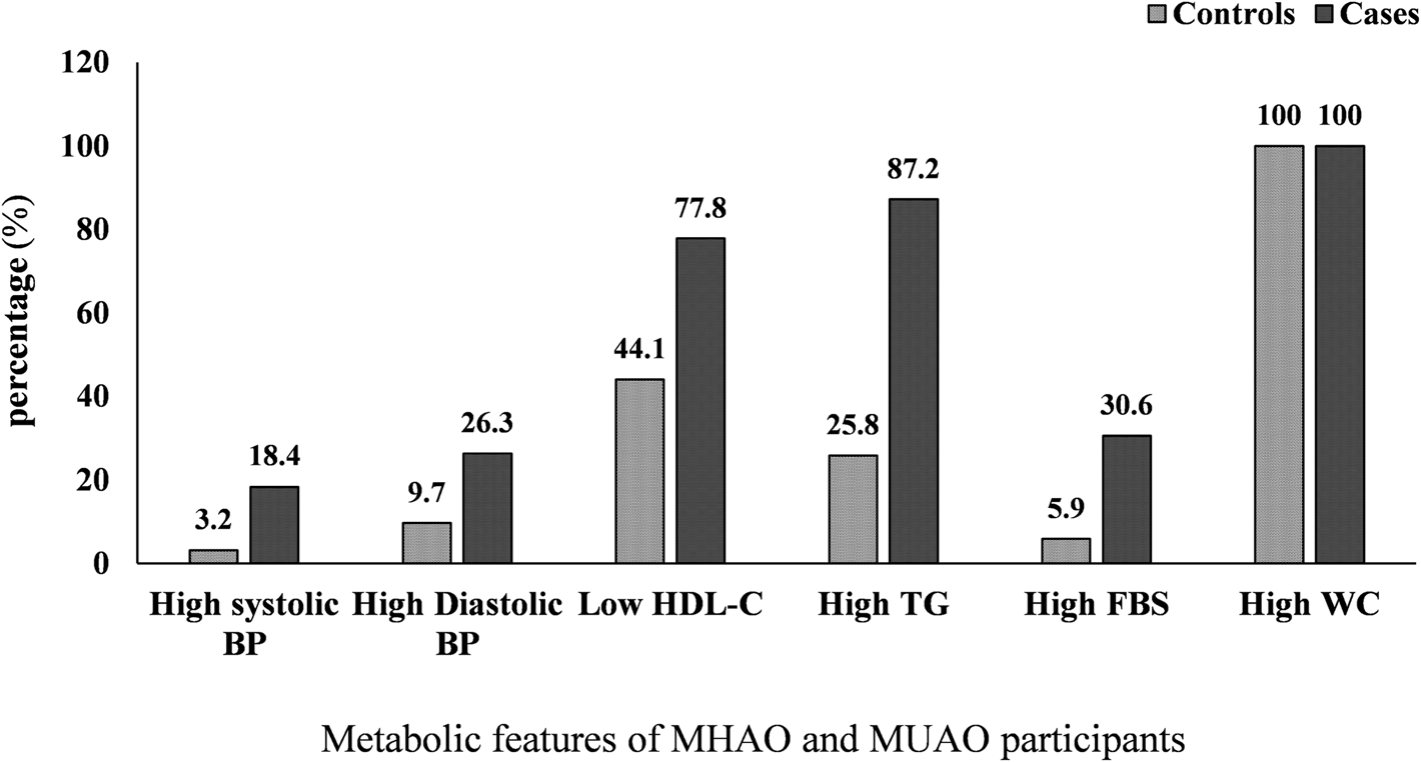
Metabolic features of MHAO and MUAO subjects. *P* < 0.001 for all except WC, using X^2^. MHAO, Metabolically Healthy Abdominally Obese; MUAO, Metabolically Unhealthy Abdominally Obese; FBS, Fasting Blood Sugar; TG, Triglycerides; HDL-C, High-Density Lipoprotein Cholesterol; Htn, Hypertension

The mean age of the participants in the case and control group was 35.14 ± 0.97 and 35.94 ± 1.13 years, respectively. Males comprised 51% of the subjects. Age, PAL, marital status, job, and level of education were not significantly different, when comparing MHAO with MUAO (Table 1). No statistically significant differences were found either in anthropometric measures or dietary intake between the two groups (Table 2). However, metabolic parameters including FBS (*p* = 0.012), TG (*p* < 0.001), and HDL-C (*p* = 0.005) were significantly different between the case and control group (Table 3). The stages of the study as a flowchart is shown in Fig. 1. Case group had higher FBS (30.6% vs. 5.9%), TG (87.2% vs. 25.8%), (systolic blood pressure (18.4% vs. 3.2%), and diastolic pressure (26.3% vs. 9.7%) than control group, Also decreased levels of HDL-C was more observed in cases than controls (77.8% vs. 44.1%). (p < 0.001 for all) (Fig. 2). Furthermore, the risk of MUAO was significantly increased with higher levels of serum FBS (OR = 7.04, 95%CI: 1.42–34.69) and TG (OR = 30.55, 95%CI: 7.48–60.67) and significantly decreased with higher HDL-C (OR = 0.22, 95% CI: 0.08–0.63) (Table 3).

**Table 1:** Demographic characteristics of the two study groups

Variable		MUAO (*n* = 36)	MHAO (*n* = 34)	*P*
Age (yrs) ^b^		35.14 ± 0.97	35.94 ± 1.13	0.599^c^
Gender (Males)		17 (54.8)	19 (48.7)	0.687^a^
Marital status	Single	4 (12.9)	5 (12.8)	0.632^a^
	Married	27 (87.1)	34 (87.2)	
BMI (kg/m^2^)	< 30	15 (48.4)	12 (30.8)	0.359^a^
	30–35	10 (32.3)	20 (51.3)	
	≥35	6 (19.4)	7 (17.9)	
Education	under diploma	7 (22.6)	7 (17.9)	0.702^a^
	Diploma	12 (38.7)	16 (41)	
	Master degree and higher	12 (38.7)	16 (41)	
PAL	Low/ moderate	16 (53.3)	21 (53.8)	0.966^a^
	High	14 (44.7)	18 (46.2)	
Job	housekeeper/ worker	21 (67.7)	24 (61.5)	0.593^a^
	Free	10 (32.3)	15 (38.5)	
	student/ clerk	14 (45.2)	12 (30.8)	
Fast food intake	Yes	10 (32.3)	13 (33.3)	0.445^£^
	No	7 (22.6)	14 (35.9)	

*MHAO* Metabolically Healthy Abdominally Obese, *MUAO* Metabolically Unhealthy Abdominally Obese, *BMI* Body Mass Index, *PAL* Physical Activity Level

Data are presented as n (%)

^a^Chi Square test

^b^Variables with normal numeric scales are reported as Mean (standard deviation)

^c^Independent Samples t-test

**Table 2:** Anthropometric and dietary parameters of the two study groups

Variable	MUAO (*n* = 36)	MHAO (*n* = 34)	*P*
Weight (kg)^a^	84.53 ± 2.48	88.29 ± 2.08	0.249 ^c^
Height (cm)^a^	164.58 ± 1.72	167.15 ± 1.89	0.323 ^c^
BMI (kg/m^2^)^a^	31.03 ± 0.67	31.68 ± 0.60	0.476 ^c^
Waist circumference (cm)^a^	105.32 ± 1.45	106.02 ± 1.12	0.701 ^c^
Hip circumference (cm)^a^	110.20 ± 1.52	110.86 ± 1.14	0.730 ^c^
Waist to hip ratio^a^	0.95 ± 0.009	0.95 ± 0.008	0.922 ^c^
Body Fat (%)^a^	32.15 ± 1.45	31.78 ± 7.02	0.845 ^c^
Fat mass (g)^a^	27.16 ± 1.49	27.88 ± 1.09	0.698 ^c^
Fat free mass (g)^a^	57.29 ± 2.04	60.58 ± 1.99	0.254 ^c^
TBW (%)^a^	41.95 ± 1.50	44.36 ± 1.45	0.253 ^c^
Energy (kcal/day) ^a^	2297.0 ± 145.29	2225.8 ± 146.44	0.731 ^c^
Dietary Carbohydrates (%)^a^	59.20 ± 1.53	58.37 ± 1.49	0.739 ^c^
Dietary Protein (%)^a^	14.25 ± 0.86	14.40 ± 0.53	0.830 ^c^
Dietary Fat (%)^a^	26.55 ± 1.42	26.53 ± 1.72	0.978 ^c^
Dietary Cholesterol (g)^b^	237.95 (138.6, 389.0)	302.90 (183.1, 464.0)	0.177^d^
Dietary SFA (g)^b^	14.87 (11.65, 28.11)	16.37 (11.31, 23.14)	0.658^d^
Dietary MUFA (g)^b^	17.17 (13.02, 31.50)	18.92 (12.12, 23.76)	0.893^d^
Dietary PUFA (g)^b^	13.42 (9.26, 19.77)	15.25 (9.96, 25.19)	0.390^d^
Dietary fiber (g)^b^	13.36 (11.50, 21.14)	14.66 (10.87–17.28)	0.804^d^

*MHAO* Metabolically Healthy Abdominally Obese, *MUAO* Metabolically Unhealthy Abdominally Obese, *BMI* Body Mass index, *PAL* Physical activity level, *TBW* Total Body Water, *SFA* Statured Fatty Acid, *MUFA* Mono Unsaturated Fatty Acid, *PUFA* Poly Unsaturated Fatty Acid

^a^Variables with normal numeric scales are reported as Mean (standard deviation)

^b^Variables with non-normal numeric scales are reported as Median (25th, 75th)

^c^ Independent Samples t- test

^d^Mann Whitney U test

**Table 3:** Biochemical characteristics in MUAO and MHAO subjects

Variables	MUAO (*n* = 36)	MHAO (*n* = 34)	OR (95% CI)^e^	*p*
FBS (mg/dL)^a^	94.86 ± 1.57	89.91 ± 1.08	7.04 (1.42–34.69)*	**0.012** ^**c**^
TG (mg/dL)^a^	223.91 ± 13.50	136.11 ± 12.20	30.55 (7.48–60.67)*	**< 0.001** ^**c**^
HDL-C (mg/dL)^a^	40.08 ± 1.15	44.61 ± 1.60	0.22 (0.08–0.63)*	**0.005** ^**c**^
SBP (mmHg)^a^	116.14 ± 2.65	111.47 ± 2.39	3.31 (0.61–17.71)	0.197 ^c^
DBP (mmHg)^a^	77.42 ± 2.57	75.00 ± 1.91	2.59 (0.71–9.42)	0.393 ^c^
IL1β (pg/mL)^b^	756 (620.2–1877.2)	710.5 (621.2–872.2)	1.02 (0.94–1.11)	0.638^d^
FFAs (nmol/L)^b^	1326.5 (1116.5–5624.7)	1292 (1148.7–2595.7)	1.01 (0.99–1.02)	0.778^d^

*MHAO* Metabolically Healthy Abdominally Obese, *MUAO* Metabolically Unhealthy Abdominally Obese, *OR* Odds Ratio, *CI* Confidence Interval, *FBS* Fasting Blood Sugar, *TG* Triglycerides, *HDL-C* High-Density Lipoprotein Cholesterol, *SBP* Systolic Blood Pressure, *DBP* Diastolic Blood Pressure, *IL1β* interleukin 1 β, *FFAs* free fatty acids

*p < 0.05

^a^ Data are reported as Mean ± standard deviation

^b^Data are reported as Median (25th, 75th)

^c^ Independent Samples t- test

^d^Mann-Whitney U test

^e^ ORs are calculated based on higher FBS, TG, Systolic PB, Diastolic BP, and lower HDL-C level

**Fig. 3:**
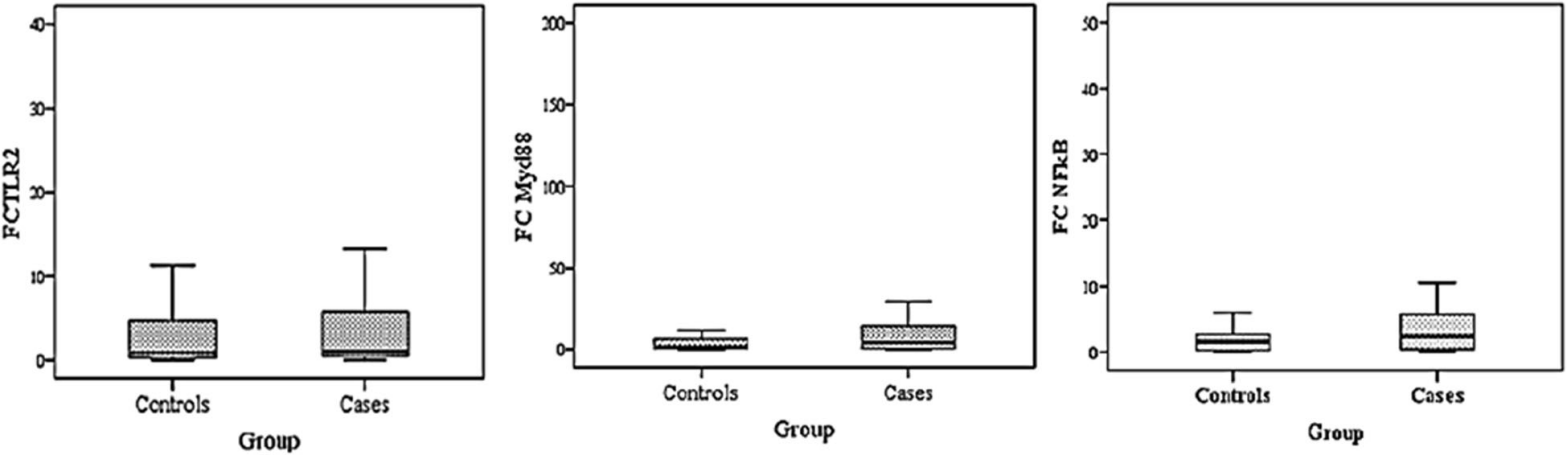
Expression ratio of TLR2, MyD88 and NFĸB in subjects with MHAO (Controls) and MUAO (Cases) *P*=*NS*. Data are presented as box plot, where boxes represent the interquartile range [IQR], the line within boxes represents the median, and the lines outside the boxes represent the lower quartile minus 1.5 times the IQR or the upper quartile plus 1.5 times the IQR. FC: Fold change, TLR2: Toll-Like Receptor 2, MyD88: Myeloid Differentiation Factor 88, NFĸB: Nuclear Factor Kappa B

**Fig. 4:**
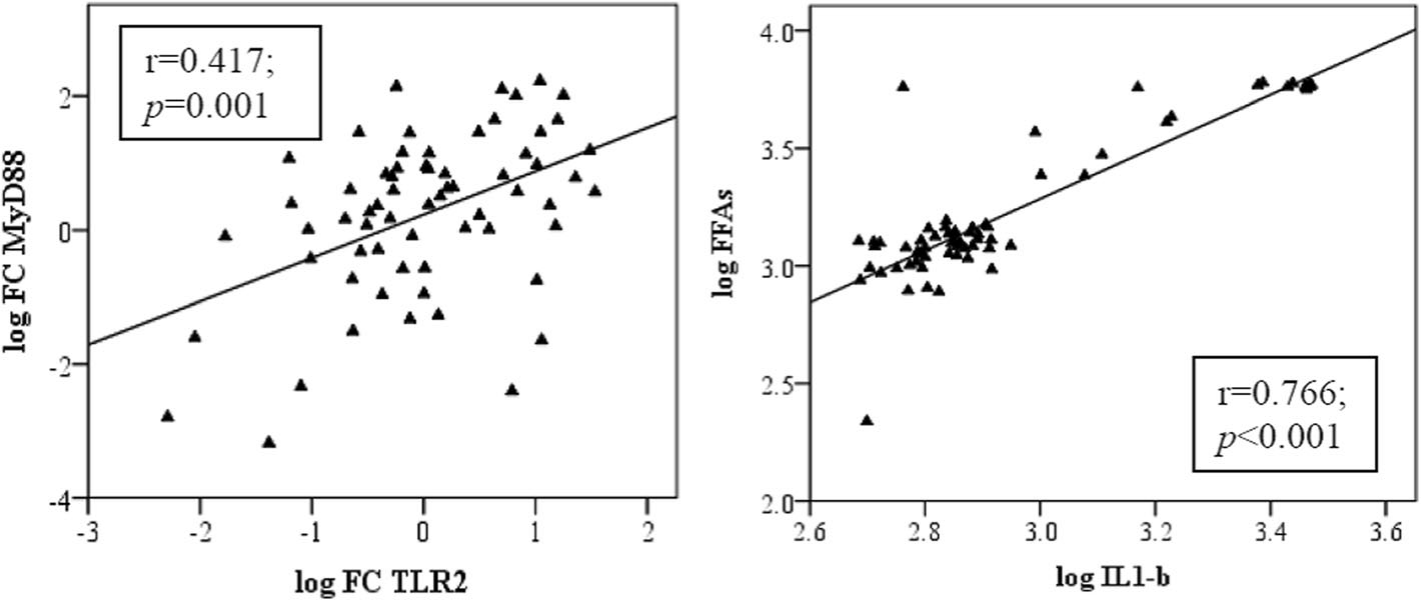
Pearson correlation between serum IL-1β and FFAs, log FC TLR2 and log FC MyD88 in the case group. IL-1β: interleukin-1 beta, FFAs: free fatty acids, FC: Fold Change, TLR2: Toll-Like Receptor 2, Myd88: Myeloid Differentiation Factor 88

There were no significant difference in the median of serum IL-1β in cases and controls (756 pg/mL, 710.5 pg/mL, respectively; *p* = 0.638). FFAs also did not show significant differences between the case and control group (1292 nmol/L*,*1326 nmol/L, respectively; *p* = 0.778) (Table 3). However, a significant correlation was found between log IL-1β and log FFAs in controls (r = 0.763; *p* < 0.001) and in cases (r = 0.760; p < 0.001) (Fig. 4). mRNA expression levels of TLR2, MyD88, and NFĸB were elevated in MUAO compared with MHAO, but it was not statistically significant (Fig. 3). The correlation of log NFĸB expression with log TLR2 (r = 0.747; *p* < 0.001) as well as log MyD88 (r = 0.747; *p* = < 0.001) was significant. Significant correlations were also noticed between log TLR2 and log MyD88 expression levels (r = 0.417; *p* < 0.001) only in the case group (Fig. 4).

## Discussion

This study for the first time focused on TLR2, MyD88, and NFĸB genes expression levels and serum levels of IL-1β and FFAs as biological indicators of the activation of these genes in MHAO vs. MUAO individuals. We found a strong association of NFĸB with TLR2 and MyD88 as well as between TLR2 and MyD88 expression levels in the PBMCs. However, the expression levels of the study genes as well as IL-1β and FFAs were similar between MUAO and MHAO groups.

As the two groups were matched on abdominal fat, it looks that gene expression levels of TLR2, MyD88 and NFĸB are highly related to abdominal obesity than to healthy or unhealthy metabolic state. There is limited data on examining the role of TLRs in metabolically healthy or unhealthy obesity and their correlations with metabolic state of obesity [26, 27] . A study showed higher mRNA expression levels of TLRs in monocytes of patients with MetS than controls after adjusting for WC [23]. In the study of Ahmad’s et al. on obese participants, the expression of TLRs and MyD88 were increased in subcutaneous adipose tissue. Migration of inflammatory PBMCs from the peripheral compartment toward the adipose tissue could be the cause of these results [29]. We used PBMCs that mostly contain monocytes and lymphocytes PBMCs are convenient for gene expression studies because they can be simply collected inadequate quantities and compared with biopsy of other tissues is less invasive [30, 31].

In line with our findings, Telle-Hansen et al. in a study on two obese groups reported no differences in the TLR2 and TLR4 gene expression in PBMCs between MHAO and MUAO groups [26]. It is notable that the significant difference was not observed even after comparison with normal-weight controls; however, in their study, the expression level of downstream adaptor proteins was not assessed. Moreover, in another cross-sectional study, the gene expression level of TLR4 and TNF-α did not differ significantly between MHAO and MUAO groups [27]. Some studies indicated that gene expression and production of IL-6 and TNFα are elevated in general and abdominal obesity [32–34]. However, such finding was not found in the present study due to similar WC in both groups. Previous studies claim that TLRs mostly signal through the adaptor protein MyD88 via activation of NFĸB [25, 35]. In our study, the expression levels of TLR2 and MyD88 were significantly correlated with NFĸB, though their expression levels were not significantly different between the two groups. Dasu et al. in a study on patients with T2DM reported that increased expression level of TLR2 and TLR4 results in raised inflammation, mediated by NFĸB [24]. However, in the case-control study of Devaraj et al. on T1DM, TLR2 and TLR4 were significantly correlated with NFĸB expression levels [25].

In another study metabolically healthy obese woman revealed lower amounts of visceral fat and a more favorable inflammatory profile in compared to the metabolically unhealthy women [11]. However, no difference was found for IL-1β with various metabolic health criteria, even after multivariate analysis and restricting the analysis to the obese (BMI ≥ 30 kg/m^2^). Another research among 58 obese postmenopausal women showed more visceral adipose tissue in women with MetS, but there were no differences in levels of inflammatory markers compared to women with no MetS [36]. These studies were carried out on obese older persons and aging may affect metabolic alterations; since the higher visceral fat region is accompanied with unhealthy metabolic phenotype in obese elderly.

Previous studies have shown that MHO definitions can influence the association between inflammatory biomarkers and MHO, based on the criteria used. We used the MHO definition of Meigs et al. in which the differences between MHO and non-MHO subjects are well-distinguished [6]. In the present study, serum levels of IL1β were not significantly different between MHAO and MUAO. Our data are in agreement with the observations from Marques-Vidal et al., who found no differences in IL-1β level of MUO subjects compared to MUO [28]. When definition was based on Meigs et al., adjustment for abdominal obesity or percent body fat did not alter the non-significant differences [6]. Moreover, their results did not show a consistent association between metabolically healthy status and IL-1β level. Based on the MHO definition used, higher, similar or lower IL-1β levels were found between MHOs and MUOs [28]. However, Jialal et al. showed that persons with MetS had significantly higher levels of inflammatory cytokines (IL-1β, IL-8, and IL-6) than control subjects without MetS [23]. In their study, it was claimed that MetS is a proinflammatory state, independent of adiposity. Conversely, ABC study indicated that visceral obesity is constantly related to higher levels of C-reactive protein (CRP) and IL-6 [37]. In another study on both subcutaneous fat and visceral fat of obese persons, unfavorable lipid profile was correlated with visceral adipocyte size, since visceral adipocyte size is directly related to a visceral fat area [38]. This may be the cause of strong association between MetS and visceral obesity than subcutaneous fat. In the present research, we failed to measure visceral fat, though WC can reflect visceral adiposity [39].

Serum FFAs result from the lipolysis of adipocytes. They are implicated in the pathogenesis of obesity-related metabolic states like IR, T2DM, and CVD [40]. *Succurro* et al. reported increased levels of plasma total FFAs in MUO compared with MHO persons [41]. Elevated serum levels of FFAs in obese individuals are usually correlated with increased amount of adipose tissue which may justify our finding regarding the similarity of serum levels of FFAs between MHAO and MUAO subjects. Boden et al. showed that FFAs link with metabolic diseases through increased generation of deleterious proinflammatory cytokines [42]. This study confirms our results which showed that FFAs are positively correlated with IL-1β levels. In fact, any augment in serum level of FFAs in the abdominally obese persons, irrespective of their metabolic aberrations, can lead to a significant increase in serum levels of IL-1β.

In this study, dietary intake was compared between the two obese groups; therefore, no significant difference was found. However, when examining the relationship of dietary parameters (intake and composition) with inflammatory markers, weak but significant associations were observed between TLR2, dietary cholesterol, and carbohydrate percent. Nevertheless, adjusting for dietary cholesterol or carbohydrate percent could not modulate the relationship between inflammatory markers and metabolic parameters. In the present study, neither FFAs nor IL1β was significantly correlated with TLR2, MyD88, and NFĸB gene expression levels. In the study of Jialal et al., FFAs were not correlated with TLR2 as well, suggesting that other factors may be involved [23].

In general, what makes our study distinct from the majority of former ones is that our groups were matched on WC to clarify the effect of abdominal obesity in contrast to the metabolic state of persons. Most of the previous studies have been carried out on patients with MetS vs. those without the syndrome or even normal weight healthy controls as well as metabolically unhealthy vs. metabolically healthy persons, without considering WC status of participants [6, 9, 11, 43]. The present study had some limitations like smaller sample size. However, any difference between the study groups was detectable due to enough power. Although the measurement of protein concentrations along with their gene expressions is more helpful, we could not do so due to budget deficit. The major strength of the current work was that the two study groups were matched based on WC.

## Conclusion

We found that WC may play a significant role as a mediator in the relation between proinflamatory genes expression levels and serum metabolic parameters. Likewise, serum levels of IL-1β and FFAs appear to be more related to abdominal obesity than to the metabolic state.

## Methods

### Study participants

In this case-control study, MUAO (*n* = 36) and MHAO controls (*n* = 34) were recruited. All participants were abdominally obese (WC ≥ 95 cm), according to the Iranian National Committee of Obesity (30) and were matched for age and gender.

Apparently healthy abdominally-obese subjects with age range of 18–60 years and BMI between 25 and 35 kg/m^2^ were included in the study. The definition of MUAO and the exclusion criteria are described in our previous works [44, 45], in detail. It should be noticed that the sample for the study was elicited from a larger population (*n* = 176) in our prior research [44], using randomization table.

### Data collection

Anthropometric indices including weight, height, and WC were measured by a trained person, using standard measurement protocol [46]. BMI was calculated by dividing weight in kilograms by the height in meters squared [46]. The standard mercury sphygmomanometer was used to measure Blood pressure (BP). The participants were relaxed and seated before measurement. After twice measurement in the left arm, the mean of two recording was considered as the BP [47]. A specific checklist was used to fill in the demographic data and medical history of each attendee. Long form of International PA questionnaire (IPAQ) was used to evaluate the physical activity [48]. A 3-day food record (two working days and one weekend) was obtained for dietary assessment and then analyzed by Nutritionist IV software (Axxya Systems, Stafford, TX), modified for Iranian foods.

### Sample size estimation

According to the study of Jialal et al. and considering TLR2 as the main variable, the effect size for TLR2 gene expression level was 7 (SD_1_ = 10 and SD_2_ = 11) [23]. Hence, sample size with α-error of 5, 80% power and a case to control ratio of 1:1 was 34 persons in each group, using the two-means formula.

### Laboratory assays

Blood samples (5 mL) were collected after 12 h overnight fast and centrifuged at 3000 rpm for 5 min to extract serum samples. FBS, TG, and HDL-C were assayed instantly, using Pars Azmoon kits (Pars Azmoon Inc., Tehran, Iran) and a Selectra 2 auto-analyzer (Vital Scientific, Spankeren, Netherlands). Inter- and intra- assay coefficient of variation (CV) were < 5% for all assays. Serum IL-1β and FFAs were analyzed after storage at − 80 °C. Serum IL-1β and FFAs levels were measured, using enzyme-linked immunosorbent assay (ELISA) (Bioassay Technology Laboratory, Shanghai Korean Biotech Co., LTD; Shanghai city, China), according to the manufacturer’s instructions. The intra-assay and inter-assay coefficients of variation were < 8 and < 10%, respectively.

### Peripheral blood mononuclear cells (PBMCs) isolation

Eight mL of fresh blood samples were collected from participants in EDTA-tubes for gene expression analysis, in the second visit under a sterile situation. PBMCs were isolated, using Ficoll-Hypaque gradient density centrifugation (Baharafshan, Tehran, Iran). Using this technique, more than 92% of cells were identified as PBMCs by flow cytometry.

### TLR2, MyD88 and NFĸB mRNA expression

RNA was extracted from PBMCs using accusol reagent (Bioneer Pacific, USA). cDNA was synthesized with Revert Aid First Strand cDNA Synthesis kit (Fermentas, Thermo fisher Scientific, USA), using random hexamer and Oilgo-dT primers. Three micrograms of RNA was utilized for cDNA synthesis. Reverse transcription was performed at 42 °C for 60 min. The first strand of cDNA was stored at − 20 °C until use for real-time PCR. Specific primers of TLR2 and MyD88 used for real-time PCR were TLR2 Fwd (5′_CTGCCTCGAGTTTCCAACACCC-3′) and TLR2 Rev. (5′_GCATTGTCCAGTGCTTCAACCTTT-3′), MyD88 Fwd (5′_GACCCA GCATTGAG GAGGATTG-3′), MyD88 Rev. (5′_AGTCGATAGTTTGTCTGTTCCAGTT-3′) NFĸB Fwd (5′_GACCGCTGCATCCACAGTTT-3′), NFĸB Rev. (5′_GGATGCGCTGACTGATAGCC-3′). Glyceraldehyde-3-phosphate dehydrogenase (GAPDH) was employed as normalize which primers were GAPDH Fwd (5′_CAAGATCATCAGCAATGCCTCC-3′) and GAPDH Rev. (5′_GCCATCA CGCCACAGTTTCC_3’). The PCR reaction mixture included 5 μl SYBR Green Mix (Takara, japan), 1 μl cDNA, 0.25 μl primer mix (4 pM), and 4.25 μl DEPC water. The PCR program initiated with preincubation step at 94 °C for 180 s, followed by 40 cycles of 94 °C for 10 s, 65 °C for 40s, and 72 °C for 20s. Reactions were performed in triplicate, using a light cycler 96 real-time PCR instrument (Roche, Switzerland). For Data analysis, the difference between average CT values of GAPDH and study gens was calculated as ΔCT in case and control groups. Then, the difference between ΔCT of gens in Case and control groups was calculated as ΔΔCT values. Then, fold change was calculated using 2^-ΔΔCT^ Equation [25, 35].

### Statistical analysis

Kolmogorov-Smirnov test was done to check the normality of data. Data are presented as mean ± standard deviation (SD) or, for skewed variables, as median (25th, 75th). Parametric data were analyzed using independent sample t-test and nonparametric data using Mann-Whitney U test. Chi-square test was applied to assess the association between two categorical variables. Spearman correlation coefficient was computed to assess the association between variables. To better represent correlation curve of TLR2, MyD88 and NFĸB, data were log transformed. Logistic regression test was used to report odds ratios and their 95% confidence intervals (CI). *P*-values less than 0.05 was considered significant. Statistical analyses was performed using SPSS software (version 17).

### Ethics approval and consent to participate

Informed written consent was obtained from each participant and the study was approved by regional ethics committee of Tabriz University of Medical Sciences, Tabriz, Iran. The whole investigation was conducted according to the principles of the Declaration of Helsinki (Ethical code: TBZMED.REC.1394.1191).

## Data Availability

The datasets used and analyzed during the current study are available from the corresponding author on reasonable request.

## Abbreviations

BMI: Body mass index
BP: Blood pressure
FBS: Fasting blood sugar
FFA: Free fatty acid
HDL-C: High-density lipoprotein cholesterol
IL-1β: Interleukin (IL)-1β
LDL-C: Low-density lipoprotein cholesterol
MetS: Metabolic syndrome
MHAO: Metabolically healthy abdominally obese
MHUO: Metabolically unhealthy abdominally obese
MyD88: Myeloid differentiation factor 88
PA: Physical activity
PBMC: Peripheral blood mononuclear cell
SFA: Saturated fatty acid
T2DM: Type 2 diabetes mellitus
TG: Triglyceride
TLR: Toll-like receptor
TNF-α: Tumor necrosis factor-α
WC: Waist circumference
